# Elevated plasma and bile levels of corisin, a microbiota-derived proapoptotic peptide, in patients with severe acute cholangitis

**DOI:** 10.1186/s13099-023-00587-4

**Published:** 2023-11-30

**Authors:** Ryo Nishiwaki, Ichiro Imoto, Satoko Oka, Taro Yasuma, Hajime Fujimoto, Corina N. D’Alessandro-Gabazza, Masaaki Toda, Tetsu Kobayashi, Hataji Osamu, Kodai Fujibe, Kenichiro Nishikawa, Tetsuya Hamaguchi, Natsuko Sugimasa, Midori Noji, Yoshiyuki Ito, Kenji Takeuchi, Isaac Cann, Yasuhiro Inoue, Toshio Kato, Esteban C. Gabazza

**Affiliations:** 1 Digestive Endoscopy Center, Doshinkai Tohyama Hospital, Minamishinmachi 17-22, Tsu, Mie 514-0043 Japan; 2 Department of Internal Medicine, Doshinkai Tohyama Hospital, Minamishinmachi 17-22, Tsu, Mie 514-0043 Japan; 3 Department of Surgery, Doshinkai Tohyama Hospital, Minamishinmachi 17-22, Tsu, Mie 514-0043 Japan; 4 Microbiome Research Center, Mie University, Edobashi 2-174, Tsu, Mie 514-8507 Japan; 5 Carl R. Woese Institute for Genomic Biology (Microbiome Metabolic Engineering), University of IL at Urbana–Champaign, Urbana, IL USA; 6 Department of Immunology, Mie University Faculty and Graduate School of Medicine, Mie University Hospital, Edobashi 2-174, Tsu, Mie 514-8507 Japan; 7 Department of Pulmonary and Critical Care Medicine, Mie University Faculty and Graduate School of Medicine, Mie University Hospital, Edobashi 2-174, Tsu, Mie 514-8507 Japan; 8 Respiratory Center, Matsusaka Municipal Hospital, Tonomachi1550, Matsusaka, Mie 515-8544 Japan; 9 Department of Gastroenterology, Matsusaka Municipal Hospital, Tonomachi1550, Matsusaka, Mie 515-8544 Japan; 10 Department of Microbiology, The University of IL at Urbana–Champaign, Urbana, IL USA; 11 Department of Animal Science, University of Illinois Urbana-Champaign, Urbana, IL 61801 USA

**Keywords:** Acute cholangitis, Choledocholithiasis, Apoptosis, Corisin, Microbiota, Dysbiosis

## Abstract

**Background:**

Acute cholangitis is a severe, life-threatening infection of the biliary system that requires early diagnosis and treatment. The Tokyo Guidelines recommend a combination of clinical, laboratory, and imaging findings for diagnosis and severity assessment, but there are still challenges in identifying severe cases that need immediate intervention. The microbiota and its derived products have been implicated in the pathogenesis of acute cholangitis. Corisin is a microbiome-derived peptide that induces cell apoptosis, acute tissue injury, and inflammation. This study aimed to evaluate the potential of plasma and bile corisin as a biomarker of acute cholangitis.

**Methods:**

Forty patients with acute cholangitis associated with choledocholithiasis or malignant disease were enrolled. Nine patients without acute cholangitis were used as controls. Corisin was measured by enzyme immunoassays in plasma and bile samples. Patients were classified into severe and non-severe groups. The associations of plasma and bile corisin with the clinical grade of acute cholangitis and other parameters were analyzed by univariate and multivariate regression analysis.

**Results:**

Plasma and bile corisin levels were significantly higher in patients with acute cholangitis than in controls. Patients with severe acute cholangitis had significantly higher plasma and bile corisin levels than those with non-severe form of the disease. Bile corisin level was significantly correlated with markers of inflammation, coagulation, fibrinolysis, and renal function. Univariate analysis revealed a significant association of bile corisin but a weak association of plasma corisin with the clinical grade of acute cholangitis. In contrast, multivariate analysis showed a significant relationship between plasma corisin level and the disease clinical grade. The receiver operating characteristic curve analysis showed low sensitivity but high specificity for plasma and bile corisin to detect the severity of acute cholangitis. The plasma and bile corisin sensitivity was increased when serum C-reactive protein level was included in the receiver operating characteristic curve analysis.

**Conclusions:**

Overall, these findings suggest that plasma and bile corisin levels may be useful biomarkers for diagnosing and monitoring acute cholangitis and that corisin may play a role in the pathophysiology of the disease by modulating inflammatory, coagulation and renal pathways.

**Supplementary Information:**

The online version contains supplementary material available at 10.1186/s13099-023-00587-4.

## Introduction

Acute cholangitis (AC) is a severe life-threatening infection of the biliary system, most commonly caused by a partial or complete blockage of the common bile duct or the common hepatic duct [1]. Biliary obstruction may be associated with gallstones, parasites, malignant disease, congenital abnormalities, primary biliary stenosis, or iatrogenic factors, including endoscopic retrograde cholangiopancreatography (ERCP) or stent placement [2]. The microbial etiology of AC is generally polymicrobial with the involvement of both aerobic and anaerobic bacteria [2]. The estimated incidence of AC in Japan is 4 to 12 per 100,000 in the population per year and may account for 1 to 3% of all hospital admissions for gastrointestinal disorders. Among patients hospitalized for gallstones, 6% to 9% are diagnosed with acute cholangitis in the United States [3]. Males and females contract the disease with similar frequency [1]. AC is a treatable condition [4]. The prognosis is generally good if adequate treatment is promptly initiated [1, 5, 6]. However, complications and fatal outcomes are common due to misdiagnosis or delayed treatment [1]. The mortality rate can be up to 40% in cases with late diagnosis and delayed treatment with antibiotics and biliary decompression [7, 8]. AC diagnosis may be difficult because the symptoms and signs are nonspecific and may overlap with other conditions, including acute pancreatitis, hepatitis, or cholecystitis [6, 9]. Therefore, AC is usually diagnosed based on a combination of clinical features, laboratory tests, and imaging studies [6]. There is currently no single clinical biomarker that can reliably confirm the diagnosis of AC.

The gut microbiota is an essential component of the human host, and it is reported to influence the metabolism, degradation, and synthesis of nutrients and the development and maintenance of an effective immune system under physiological conditions [10]. However, the gut microbiota and its dysfunction may also contribute to the pathogenesis of hepatobiliary diseases, including AC [11]. For example, the gut microbiota may be a source of microbes in AC. Bacteria that normally inhabit the gut microbiota, such as *Escherichia coli*, *Klebsiella pneumoniae*, and *Enterococcus faecalis*, are the most common bacteria isolated from the bile cultures of AC patients [2, 12, 13]. Consistent with this observation, a recent study using 16S rRNA gene sequencing showed that the bile and duodenal microbial communities are similar in patients with choledocholithiasis, which is the most frequent cause of AC [4, 14, 15]. Dysbiosis of the gut and bile microbial flora and abnormal metabolite profiles reported in patients with biliary disease further support the potential pathogenic role of microbiota in AC [16–20]. Bacteria may enter the biliary system by duodenal-biliary reflux or by hematogenous or lymphatic bacterial spread [1]. The most common mechanism seems to be the reflux of duodenal microbial content into the common bile duct due to increased intrabiliary pressure [21, 22]. The increased ductal pressure caused by a partial or intermittent obstruction of the bile duct leads to dysfunction of the sphincter of Oddi, which consequently cannot properly close, allowing reflux of duodenal microbial flora [23].

The direct involvement of the gut microbiota in the pathogenesis of AC suggests that factors released by microbes may serve as good disease biomarkers. We recently reported that a peptide that induces cell apoptosis, named corisin, is released by the lung microbiota and is elevated in the serum and bronchoalveolar lavage fluid from patients and experimental animal models with acute lung injury [24–26]. Apoptosis is a form of programmed cell death characterized by membrane blebbing, chromatin condensation, DNA fragmentation, and the formation of apoptotic bodies [27]. A large body of evidence has shown that apoptosis plays an important role in several physiological processes and the pathophysiology of human diseases, including disorders of the biliary system [27, 28]. In the present study, we evaluated the potential usefulness of corisin as a biomarker in AC patients.

## Materials and methods

### Subjects

**Fig. 1:**
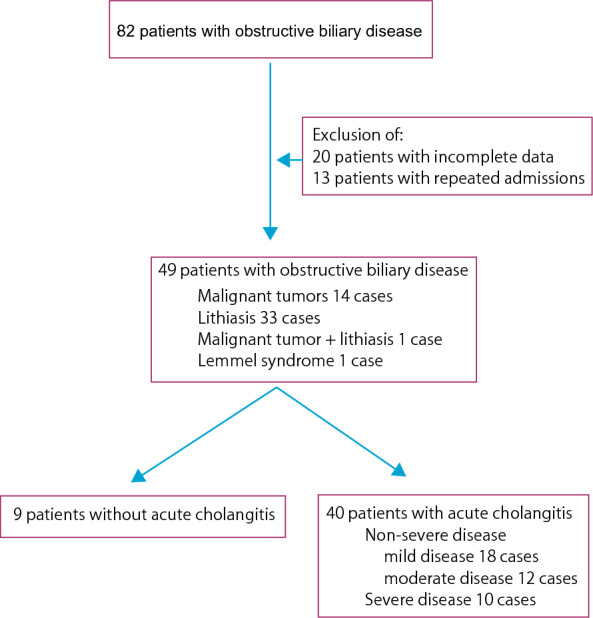
Flow chart for patients’ selection. Eighty-two patients with obstructive biliary disease underwent endoscopic retrograde cholangiopancreatography. Of these, 49 patients were included in the study

This is a prospective study that included 40 patients (mean age 80.15 ± 1.71 years old) diagnosed with AC and underwent endoscopic retrograde cholangiopancreatography (ERCP) at Doshinkai Tohyama Hospital from March 2022 to February 2023 (Fig. 1). There were 24 males and 16 females. AC was diagnosed according to the diagnostic criteria of the 2018 Tokyo Guidelines. The Tokyo Guidelines provide criteria for diagnosing acute cholangitis based on clinical features, laboratory data, and imaging findings [6, 29, 30]. They also provide a severity grading system that classifies acute cholangitis into mild (grade 1), moderate (grade 2), and severe (grade 3) categories based on the presence or absence of organ dysfunction, cholangitis-related symptoms, and inflammatory response [6, 29, 30]. The inclusion criteria in the present study were suspicion of AC, obtention of written informed consent, and availability of ERCP study. The exclusion criteria were severe comorbidity that may affect the safety of the study, failure to collect bile and blood samples, and virus-related hepatitis. Data from 9 patients (mean age 72.89 ± 6.03; 3 males and 6 females) who underwent ERCP because of biliary obstruction but without AC were used as control.

### Diagnosis

All patients underwent clinical evaluation, physical examination, routine hemogram, and biochemical tests. Patients with suspicion of biliary obstruction underwent additional diagnostic procedures, including computed tomography. The diagnosis and severity grading of AC followed the diagnostic criteria of the 2018 Tokyo Guidelines. Blood was sampled during admission, and bile was collected during the ERCP study. The samples were centrifuged, and the plasma and bile supernatant were stored at − 80 °C until use. A portion of the bile sample was sent to the laboratory for microbial culture.

### ERCP procedure

An expert endoscopist performed the ERCP using a standard therapeutic duodenoscope (Olympus TJF-290 V; Olympus, Tokyo, Japan). Guidewires (Olympus VISIGlide2), a catheter (Swish; Boston Scientific, Marlborough, MA), a plastic stent (flexima size 7Fr; 5, 7 or 10 cm; Boston Scientific, Marlborough, MA), and a biliary dilation balloon (hurricane size 8 mm; Boston Scientific, Marlborough, MA) were also used. The patients were asked to avoid meals and fast on the day of the endoscopic procedure from 6:00 AM. They received sedation with midazolam 4 mg and pentazocine 7.5 mg or 15 mg. All patients received antibiotics (cefmetazole 1 g twice or meropenem 0.5 g three times) before and after the ERCP procedure. Cannulation into the common bile duct was performed through the major duodenal papilla for contrast injection. The guidewire and plastic stent were then placed. Patients with lithiasis underwent endoscopic sphincterotomy, and the stones were extracted from the common bile duct using a stone basket or stone balloon. Some patients underwent endoscopic papillary small balloon dilation or large balloon dilation. A metal stent was introduced into the common bile duct in some cases of malignant tumors.

### Laboratory analysis

All patients underwent routine hemograms and biochemical tests at the Clinical Laboratory of Doshinkai Tohyama Hospital. The following parameters were measured using standard laboratory methods: white blood cells, platelet count, total protein, albumin, hemoglobin, total bilirubin and direct bilirubin, aspartate aminotransferase, alanine transaminase, γ-glutamyl transpeptidase, amylase, alkaline phosphatase, electrolytes, procalcitonin and C-reactive protein, creatinine, blood urea nitrogen, activated partial thromboplastin time, prothrombin time, and fibrin-degradation products. The levels of tumor necrosis factor-α, interleukin-6, and FasL were measured using commercially available immunoassays according to the manufacturer's instructions (R&D Systems, Minneapolis, MN). The DNA was extracted from the bile samples, as previously described [31]. Sequencing of the 16S rDNA, microbial composition analysis, microbial diversity assessment, and taxonomic assignment were conducted at Macrogen Japan Corporation (Tokyo, Japan).

### Measurement of corisin in serum and bile samples

We measured corisin using an in-house enzyme immunoassay we developed and described previously with some modifications [25]. In brief, a polyclonal anti-transglycosylase antibody we previously developed [25] was diluted in phosphate-buffered saline (PBS) and coated onto a 96-well plate at a concentration of 2.5 µg/mL (100 µL/well). The plate was incubated for 18 h at 4 °C. The plate wells were washed five times with PBS, then a blocking buffer was added to each well (150 µL/well). The plate was incubated for 2 h at room temperature. After washing four times with PBS, 100 µL of standard concentrations of synthetic corisin or tenfold diluted serum or bile samples were added to each well. The plate was sealed and incubated for 18 h at 4 °C. The samples were discarded, and the wells were washed four times with PBS. After washing, 100 µL of the biotin-labeled monoclonal anticorisin antibody we previously developed [25] was diluted at a concentration of 2.5 µg/mL was added to each well. The plate was incubated at room temperature for 2 h. After appropriate washing, 100 µL of diluted (1/2000) horseradish peroxidase-labeled streptavidin was added to each well. The plate was incubated at room temperature for 1 h. After appropriate washing, color development was performed. Absorbance was measured at 450 nm using a microplate reader (BIOD-RAD iMark™, Hercules, CA, USA). The concentrations of corisin were extrapolated from a curve created using standard concentrations of corisin. The inter- and intra-assay coefficients of variation were less than 10%.

### Statistical analysis

Data are expressed as the mean ± the standard errors of the mean (SEM). The Kolmogorov–Smirnov and Shapiro–Wilk tests were used to analyze the normality of the data. The statistical difference between two variables was analyzed by the Mann–Whitney U test and among three variables by analysis of variance with Fisher's predicted least significant difference test. The strength of the correlation between factors was assessed by Spearman's correlation. A continuous variable representing the severity of AC was created with four categories: grade 0 (no AC), grade 1 (mild AC), grade 2 (moderate AC), and grade 3 (severe AC), and univariate and multivariate regression analyses were performed to identify the predictors of AC severity. Receiver operating characteristic (ROC) curve analysis was conducted to evaluate the diagnostic performance of the variables for AC severity. Graph-pad Prism version 9.0 (San Diego, CA, USA) was used for statistical analysis. A p < 0.05 was considered statistically significant.

## Results

### Causes of biliary obstruction and AC

The cause of biliary obstruction among AC patients was lithiasis in 28 cases, malignant tumors in 10, Lemmel syndrome in 1, and lithiasis plus malignant tumor in 1 case. The underlying disease of AC patients included lithiasis (29 cases), type 2 diabetes mellitus (8 cases), cholangiocarcinoma (6 cases), pancreatic cancer (2 cases), chronic kidney disease (2 cases), stroke sequelae (2 cases), Lemmel syndrome (1 case), and cancer of the thyroid, rectum, prostate, duodenal papilla, breast, and liver (1 case each). The cause of biliary obstruction among non-AC patients was lithiasis (5 cases) and malignant tumor (4 cases). The underlying disease of non-AC patients was lithiasis (5 cases), pancreatic cancer (3 cases), and cancer of the lung and breast (1 case). Nine patients from the AC group and two from the non-AC group had a history of sphincterotomy. The AC patients were categorized into two groups based on disease severity: 30 with non-severe AC (18 with mild AC, 12 with moderate AC), and 10 with severe AC (Table 1).

**Table 1:** Clinical profile of the patients

Variable	Non-AC group	AC group	All subjects
No of subjects	9	40	49
Age (years-old ± SEM)^a^	72.89 ± 6.03	80.15 ± 1.71	78.82 ± 1.79)
Sex			
Males	3 (33.3%)	24 (60%)	27 (55.1%)
Females	6 (66.7%)	16 (40%)	22 (44.9%)
Underlying diseases			
Biliary lithiasis	5 (55.6%)	30 (75%)	35 (71.4%)
Type 2 diabetes mellitus	4 (44.4%)	8 (20.0%)	12 (24.5%)
Stroke sequalae	0 (0%)	2 (5.0%)	2 (4.1%)
Chronic kidney disease	1 (11.1%)	2 (5.0%)	3 (6.1%)
Lemmel syndrome	0 (0%)	1 (2.5%)	1 (2.0%)
Malignant tumors			
Cholangiocarcinoma	0 (0%)	4 (10.0%)	4 (8.1%)
Pancreatic cancer	2 (22.2%)	1 (2.5%)	3 (6.1%)
Duodenal papillary cancer	0 (0%)	1 (2.5%)	1 (2.0%)
Cholangiocarcinoma + pancreatic cancer	0 (0%)	1 (2.5%)	1 (2.0%)
Hepatocellular carcinoma + gastric cancer	0 (0%)	1 (2.5%)	1 (2.0%)
Lung cancer	1 (11.1%)	0 (0%)	1 (2.0%)
Pancreatic cancer + breast cancer	1 (11.1%)	1 (2.5%)	2 (4.1%)
Thymoma	0 (0%)	1 (2.5%)	1 (2.0%)
Rectal carcinoma	0 (0%)	1 (2.5%)	1 (2.0%)
Cholangiocarcinoma + prostate cancer		1 (2.5%)	1 (2.0%)
Causes of biliary obstruction			
Lithiasis	5 (55.6%)	29 (72.5%)	34 (69.3%)
Malignant tumor	4 (44.4%)	9 (22.5%)	13 (26.5%)
Lithiasis + malignant tumor	0 (0%)	1 (2.5%)	1 (2.0%)
Lemmel syndrome	0 (0%)	1 (2.5%)	1 (2.0%)
History of sphincterotomy	2 (22%)	9 (13%)	11 (23%)
Grade of AC			
Grade I (mild)		18 (45%)	18 (36.7%)
Grade II (moderate)		12 (30%)	12 (24.5%)
Grade III (severe)		10 (25%)	10 (20.4%)

AC acute cholangitis

^a^Data are the mean ± standard errors of the means

**Fig. 2:**
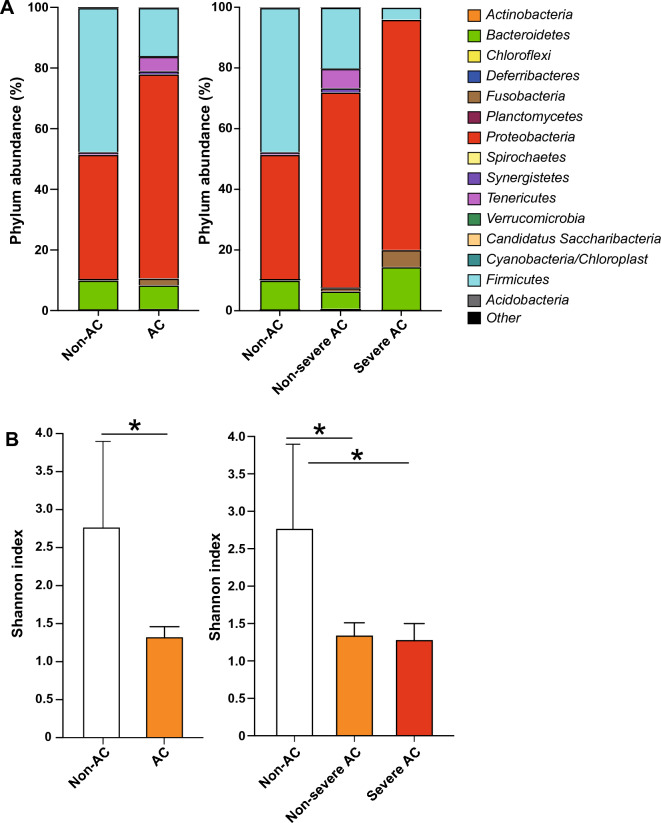
Phylum abundance and Shannon diversity. Microbial composition analysis (**A**) and microbial diversity (**B**) assessment were performed as described under Materials and Methods. *p < 0.05. Non-AC, non-acute cholangitis patients; AC, acute cholangitis patients

No significant difference was observed in any parameter between patients grouped by cause of biliary obstruction (Additional file 1: Table S1). The most common microorganisms isolated from the bile culture were *Escherichia coli* in 24 cases, *Klebsiella pneumoniae* ssp. *pneumoniae* in 18 cases, and *Enterococcus* in 13 cases (Additional file 1: Table S2). The bile microbial community analysis at the phylum level revealed a significant reduction in the abundance of *Firmicutes* in AC patients compared to the non-AC group, while *Fusobacteria* was markedly enriched in patients with severe AC compared to non-AC and non-severe patients. No significant differences between patient groups were observed in the abundance of other bacterial phyla, including *Proteobacteria* and *Bacteroidetes* (Fig. 2A). At the genus level, the abundance of *Serratia* and *Lactobacillus* was significantly lower in the AC group than in the non-AC group, but there was no significant difference between non-severe and severe patients. The abundance of *Fusobacterium*, *Escherichia*/*Shigella*, and *Bacteroides* was significantly higher in patients with severe AC than in patients with non-severe AC (Additional file 1: Fig. S1). The Shannon diversity was significantly lower in all AC patients and both non-severe and severe AC subgroups than in the non-AC group (Fig. 2B).

### Increased plasma and bile concentrations of corisin in AC patients

**Fig. 3:**
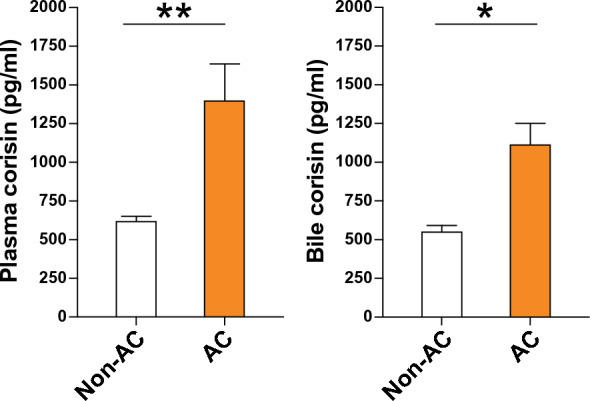
Increased levels of plasma and bile corisin in patients with acute cholangitis. Corisin was measured by enzyme immunoassays. There were 40 patients with acute cholangitis and 9 without acute cholangitis. Data are expressed as the mean ± SEM. Statistical analysis was performed by the Mann–Whitney U test. AC, acute cholangitis. *p < 0.05; **p < 0.01

We measured the concentration of corisin in plasma and bile from patients with and without AC using enzyme immunoassay and compared the results. Plasma and bile levels of corisin were significantly higher in patients with AC than those without AC (Fig. 3).

Evaluation of routine laboratory parameters showed that patients with AC also had higher blood levels of the inflammatory markers CRP and IL-6, increased fibrinolysis activity as indicated by FDP, and shorter prothrombin time than patients without AC. The renal function marker serum creatinine was significantly higher in patients with AC than those without AC (Table 2).

**Table 2:** Clinical and laboratory profile of all subjects

	Normal values	Non-AC group	AC group	P values
No of subjects		9	40	
Systolic pressure	< 120 mmHg	126.89 ± 8.7	128.60 ± 3.49	0.36
Diastolic pressure	< 80 mmHg	76.73 ± 3.43	69.28 ± 1.94	0.04
Body temperature	36.1–37.2 °C	36.97 ± 0.20	36.87 ± 0.11	0.34
White blood cells	3,300–8,600/µL	8.05 ± 1.53	10.25 ± 0.80	0.08
Blood platelets	13.8–30.9 × 10^4^ (µL)	230.80 ± 31.88	178.60 ± 12.74	0.07
Blood hemoglobin	11.6–16.5 g/dL	12.43 ± 0.61	11.68 ± 0.34	0.22
Blood total protein	6.5–8.0 g/dL	6.45 ± 0.29	6.45 ± 0.11	0.45
Blood albumin	4.0–5.2 g/dL	3.37 ± 0.23	3.09 ± 0.11	0.07
Blood total bilirubin	0.2–1.2 mg/dL	3.17 ± 0.88	3.40 ± 0.40	0.33
Blood direct bilirubin	0–0.3 mg/dL	2.12 ± 0.74	2.15 ± 0.42	0.49
Serum aspartate aminotransferase	≤ 30 U/L	176.20 ± 55.71	204.30 ± 36.52	0.46
Serum alanine transaminase	≤ 30 U/L	191.1 ± 53.2	155.20 ± 22.30	0.28
Serum γ-glutamyl transpeptidase	≤ 50 U/L	391.0 ± 138.2	382.8 ± 51.02	0.40
Serum alkaline phosphatase	38–113 U/L	272.70 ± 39.60	353.7 ± 46.57	0.44
Serum amylase	43–116 U/L	97.63 ± 29.56	82.75 ± 11.69	0.45
Serum Na	135–147 mmol/L	138.20 ± 1.61	137.5 ± 0.66	0.28
Serum K	3.3–4.8 mmol/L	4.04 ± 0.22	4.02 ± 0.09	0.31
Serum Cl	98–108 mmol/L	100.7 ± 2.42	100.1 ± 2.42	0.31
Serum procalcitonin	< 0.05 ng/mL	1.56 ± 1.20	5.66 ± 2.03	0.07
Serum C-reactive protein	≤ 0.3 mg/dL	4.01 ± 1.77	9.97 ± 1.67	0.02
Blood urea nitrogen	7–24 mg/dL	18.10 ± 2.81	26.12 ± 3.56	0.37
Serum creatinine	≤ 0.70 mg/dL	1.40 ± 0.69	1.49 ± 0.25	0.04
Activated partial thromboplastin time	24–39 s	34.84 ± 4.43	35.82 ± 1.43	0.16
Prothrombin time (sec)	9–13 s	100.4 ± 11.81	80.92 ± 4.32	0.03
Plasma fibrin-degradation products	< 5 µg/mL	5.35 ± 1.24	15.77 ± 3.09	0.04
Peripheral oxygen saturation	95–100%	97.44 ± 0.37	96.73 ± 0.40	0.15
Plasma IL-6	< 17.4 pg/mL	178.20 ± 21.30	3088.0 ± 1294	0.02
Plasma TNFα	0–2.2 pg/mL	288.1 ± 12.9	551.4 ± 109.6	0.48
Plasma FasL	0.2–1.5 pg/mL	62.13 ± 6.36	70.16 ± 5.94	0.20
Bile IL-6	< 10 pg/mL	259.70 ± 95.02	819.90 ± 299.60	0.05
Bile TNFα (pg/mL)	< 5 pg/mL	374.70 ± 72.79	671.60 ± 122.80	0.13
Bile FasL (pg/mL)	0.5–2.5 pg/mL	115.50 ± 9.99	132.40 ± 18.25	0.17

Data are expressed as the mean ± standard error of the mean

TNF-α, tumor necrosis factor-α; IL-6, interleukin-6; AC, acute cholangitis

These results suggest that the microbiota from patients with AC have an enhanced systemic and biliary expression of corisin in association with an increased inflammatory response, altered coagulation activation, and impaired renal function.

### Increased plasma and bile corisin levels with clinical progression of AC

**Fig. 4:**
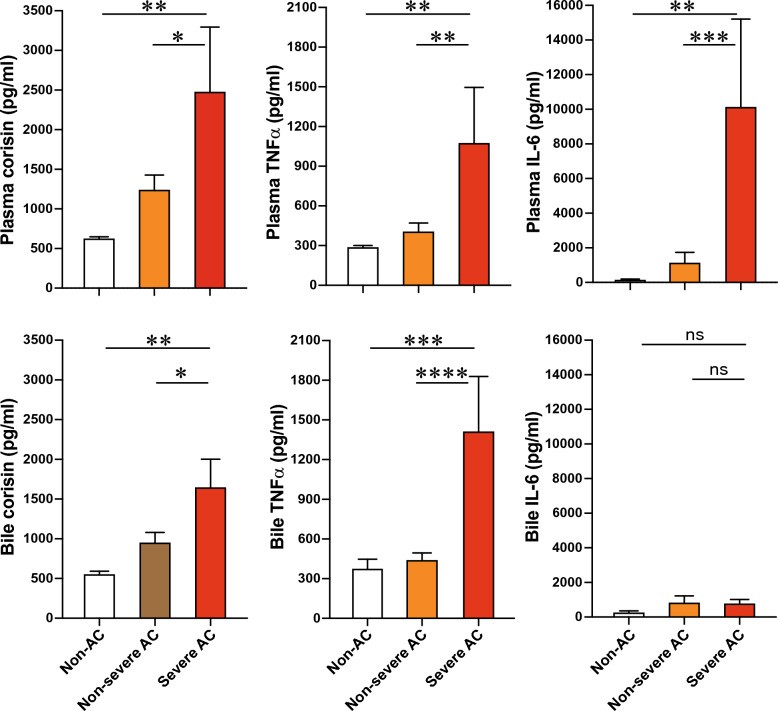
Increased levels of plasma and bile corisin in patients with severe acute cholangitis. Corisin was measured by enzyme immunoassays. There were 9 patients with non-cholangitis, 30 with non-severe (mild disease 18 cases + moderate disease 12 cases) acute cholangitis, and 10 with severe disease. Data are expressed as the mean ± SEM. Statistical analysis was performed by ANOVA Fisher’s predicted least significant difference test. *p < 0.05; **p < 0.01; ***p < 0.001; ****p < 0.0001

The plasma and bile levels of corisin were compared between patients with severe AC, non-severe (mild and moderate) AC, and non-AC patients. Patients with severe AC had significantly higher plasma corisin concentrations than non-AC and non-severe AC patients. The bile corisin level was significantly elevated in patients with severe AC compared to non-severe AC and non-AC patients (Fig. 4).

The plasma levels of TNFα and IL-6 were significantly increased in severe AC patients compared to non-severe and non-AC patients. The bile level of TNF-α but not that of IL-6 was significantly increased in patients with severe AC compared to non-severe AC and non-AC patients (Fig. 4). In addition, patients with severe AC had significantly increased white blood cell count, significantly reduced platelet count, and significantly elevated concentrations of CRP, FDP, procalcitonin, blood urea nitrogen, and creatinine compared to non-severe and non-AC patients (Table 3).

**Table 3:** Clinical profile and laboratory data by disease severity

	Non-AC	Non-severe AC	Severe AC
No of subjects	9	30	10
Age (years-old ± SEM)	72.89 ± 6.03	80.70 ± 2.06	78.50 ± 3.02
Systolic pressure (mmHg)	126.80 ± 8.07	131.03 ± 3.25	120.60 ± 9.99
Diastolic pressure (mmHg)	75.80 ± 3.65	70.97 ± 2.06	64.20 ± 4.53
White blood cells (103/µL)	8.05 ± 1.53	9.22 ± 0.73	13.33 ± 2.13*†
Blood platelets (104/µL)	230.80 ± 31.88	200.00 ± 13.64	114.20 ± 20.09*†
Blood hemoglobin (g/dL)	12.43 ± 0.61	11.76 ± 0.40	11.44 ± 0.67
Blood total protein (g/dL)	6.45 ± 0.29	6.50 ± 0.13	6.31 ± 0.17
Blood albumin (g/dL)	3.37 ± 0.23	3.11 ± 0.13	3.04 ± 0.18
Blood total bilirubin (mg/dL)	3.17 ± 0.88	3.03 ± 0.43	4.48 ± 0.94
Blood direct bilirubin (mg/dL)	2.12 ± 0.74	1.66 ± 0.37	4.22 ± 1.30
Serum aspartate aminotransferase (U/L)	176.20 ± 55.71	210.60 ± 43.55	185.10 ± 68.73
Serum alanine transaminase (U/L)	191.10 ± 53.20	146.90 ± 23.43	180.20 ± 56.77
Serum γglutamyl transpeptidase (U/L)	391.00 ± 138.20	379.90 ± 62.07	391.20 ± 89.97
Serum alkaline phosphatase (U/L)	272.70 ± 39.60	334.20 ± 53.91	412.10 ± 94.80
Serum amylase (U/L)	97.63 ± 29.56	76.87 ± 13.11	100.4 ± 25.67
Serum Na (mmol/L)	138.20 ± 1.61	138.00 ± 0.67	135.90 ± 166
Serum K (mmol/L)	4.04 ± 0.22	4.01 ± 0.08	4.06 ± 0.28
Serum Cl (mmol/L)	100.70 ± 2.42	100.30 ± 3.18	99.70 ± 1.86
Serum procalcitonin	1.56 ± 1.20	2.63 ± 1.22	17.34 ± 7.53*†
Serum C-reactive protein (mg/dL)	4.01 ± 1.77	6.75 ± 1.36	20.73 ± 4.07*†
Blood urea nitrogen (mg/dL)	18.10 ± 2.81	16.55 ± 1.22	54.85 ± 9.13*†
Serum creatinine (mg/dL)	1.40 ± 0.69	0.86 ± 0.04	3.38 ± 0.78*†
Activated partial thromboplastin time (sec)	34.84 ± 4.43	36.02 ± 1.77	35.17 ± 2.07
Prothrombin time (sec)	100.40 ± 11.81	81.86 ± 5.20	77.89 ± 7.62
Plasma fibrin-degradation products	5.35 ± 1.24	12.44 ± 3.08	26.99 ± 7.77*†
Peripheral oxygen saturation (%)	97.44 ± 0.37	96.63 ± 0.51	97.00 ± 0.47

Data are expressed as the mean ± standard error of the mean

AC, acute cholangitis; SEM, standard error of the means; TNF-α, tumor necrosis factor-α; IL-6, interleukin-6

*p < 0.5 vs non-AC; ^†^p < 0.01 vs non-severe AC

These results suggest that the clinical progression of AC is associated with increased release of corisin, enhanced inflammatory response, abnormal fibrinolysis, and renal dysfunction.

### Significant correlation of bile corisin with inflammatory, coagulation, and renal function markers

**Fig. 5:**
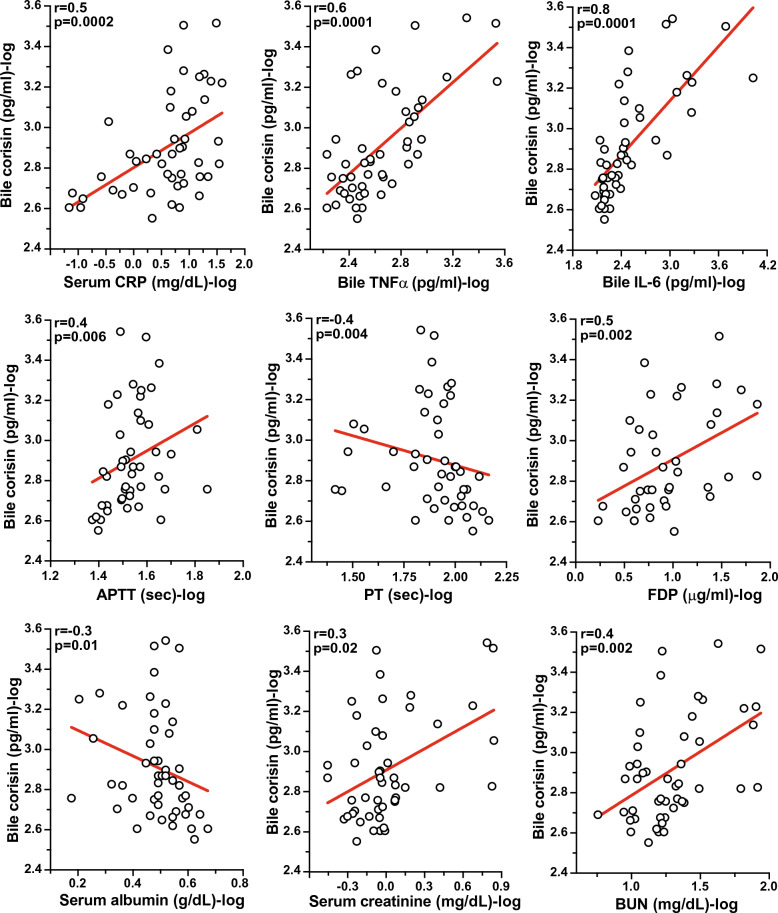
Significant correlation of bile corisin with inflammatory, coagulation, fibrinolysis, and renal function markers. Corisin was measured by enzyme immunoassays. Patients with (n = 40) and without (n = 9) acute cholangitis were included to evaluate the correlation. Statistical analysis was performed by Spearman correlation

The bile concentration of corisin was significantly and positively correlated with the inflammatory markers CRP, TNF-α, and IL-6, as well as with the coagulation/fibrinolysis markers APTT and FDP. Corisin levels were also significantly and positively correlated with the markers of renal function creatinine and blood urea nitrogen (BUN). In addition, the bile level of corisin was significantly and negatively correlated with the prothrombin time and serum albumin level (Fig. 5). However, the plasma level of corisin was not significantly correlated with any inflammatory, coagulation, or renal function parameters.

These results suggest that the local generation of corisin in bile is associated with systemic markers of inflammation and coagulation abnormalities.

### Predictors of the clinical grade of AC

**Fig. 6:**
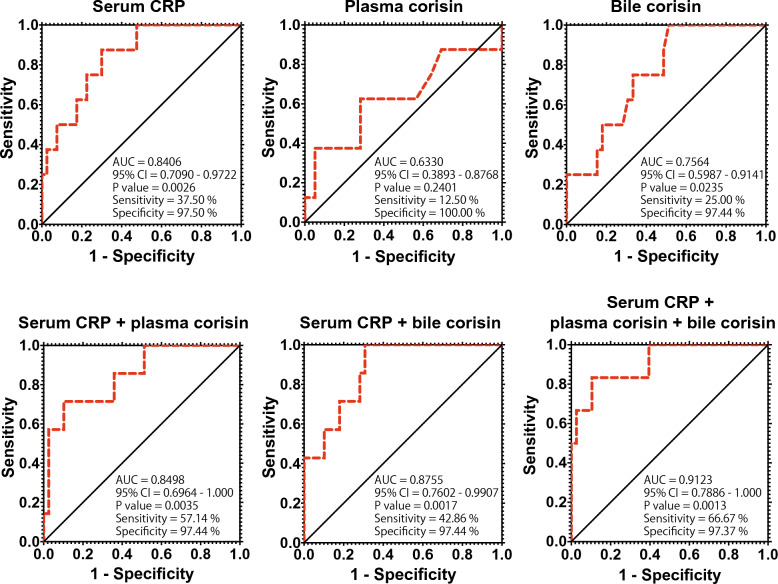
Receiver operating characteristic (ROC) curve analysis for the diagnosis of severe acute cholangitis. The ROC curves of serum C-reactive protein (CRP), plasma corisin, and bile corisin (A) or their combination (B) were plotted to evaluate their diagnostic performance. AUC, area under the curve

Based on the 2018 Tokyo Guidelines classification of AC severity, patients were grouped into grades 1 (mild AC), 2 (moderate AC), and 3 (severe AC). Patients with biliary obstruction but without AC were grouped as grade 0. Univariate and multivariate regression analysis was conducted to assess the value of several variables in predicting AC severity, using the clinical grade of AC as a continuous dependent variable. In the univariate analysis, the following variables were significantly associated with the grade of disease: blood platelet count, direct bilirubin level, C-reactive protein level, and prothrombin time. Multivariate regression analysis was then conducted for variables with p < 0.1 in the univariate analysis. The blood levels of C-reactive protein and corisin were significantly associated with the clinical grade of AC in the multivariate analysis (Table 4). The ROC curve analysis showed that the area under the curve (AUC) was significantly increased for serum CRP and bile corisin but not for plasma corisin, compared to a baseline value (Fig. 6). This suggests that serum CRP and bile corisin may be more accurate predictors of AC severity than plasma corisin. However, the specificity for detecting AC severity was high, but the sensitivity was low for CRP and plasma and bile corisin, suggesting that these tests may be better at identifying patients with severe AC than mild or moderate AC. The sensitivity improved when the serum CRP was combined with plasma and bile corisin in the ROC curve analysis, suggesting that combining these tests may improve the overall accuracy of predicting AC severity (Fig. 6).

**Table 4:** Univariate and multivariate analysis of predictors of disease clinical grade

Variables	β	95% Confidence interval	P values
Univariate analysis			
Body temperature	− 0.06840	− 0.4893 to 0.3525	0.7452
Blood platelets	− 0.005229	− 0.008410 to − 0.002048	0.0018
Blood total protein	− 0.1432	− 0.5465 to 0.2600	0.4784
Blood albumin	− 0.3704	− 0.7847 to 0.04396	0.0786
Blood direct bilirubin	0.1473	0.02161 to 0.2730	0.0228
Serum aspartate aminotransferase	− 0.0004470	− 0.001809 to 0.0009148	0.5123
Serum alanine transaminase	− 0.0004057	− 0.002493 to 0.0011681	0.6975
Serum γ-glutamyl transpeptidase	0.0004206	− 0.0004873–0.001329	0.3559
Serum amylase	− 0.001653	− 0.005628 to 0.0022323	0.4070
Serum C-reactive protein	0.05997	0.03570 to 0.08423	< 0.0001
Serum creatinine	0.2115	0.04711 to 0.3759	0.0128
Activated partial thromboplastin time	0.005191	− 0.02722 to 0.03760	0.7484
Prothrombin time	− 0.01032	− 0.2055–0.00007634	0.0484
Peripheral oxygen saturation	0.01428	− 0.008877 to 0.03744	0.2209
Plasma corisin	0.0001820	0.00004644 to 0.0004104	0.1156
Bile corisin	0.0004046	0.00002717 to 0.0007820	0.0362
Multivariate analysis			
Blood platelets	− 0.002166	− 0.005095 to 0.0007623	0.1413
Blood albumin	0.04396	− 0.3469 to 0.4348	0.8199
Blood direct bilirubin	0.03063	− 0.09576 to 0.1570	0.6242
Serum C-reactive protein	0.04274	0.01417 to 0.07131	0.0047
Serum creatinine	− 0.1259	− 0.3268 to 0.07495	0.2103
Prothrombin time	− 0.006219	− 0.01510 to 0.002658	0.1628
Plasma corisin	0.0001760	0.000004075–0.0003480	0.0451
Bile corisin	0.0002167	− 0.0002312 to 0.0006646	0.3310

## Discussion

The present study found that elevated circulating and bile levels of the microbiome-derived pro-apoptotic peptide corisin are correlated with clinical progression in AC patients.

The biliary microbiome has been the focus of recent studies. Traditional culture techniques suggested that the biliary tract was sterile, but metagenomic analysis using pyrosequencing of bacterial 16S rRNA genes revealed a diverse bacterial population in the biliary system [20, 32, 33]. This microbial population seems to originate from the gut, and its imbalance or dysbiosis has been associated with inflammatory and malignant biliary diseases [11, 20, 34, 35]. Some evidence for this link is the presence of bacteria (e.g., *Helicobacter bilis*) in gallstones, the production of autoantibodies against biliary epithelial cell proteins that have molecular mimicry with bacterial proteins in primary biliary cirrhosis or primary sclerosing cholangitis, and the overgrowth of specific enteric bacteria in cholangiocarcinoma [16, 36–39]. The microbiota is also involved in biliary tract bacterial infections. A previous report demonstrated that the bile and gut of patients with acute cholecystitis are enriched with a similar family of bacteria, indicating that the microbiota of the biliary system and gut interact bidirectionally and may serve as a source of pathogens [40]. Apart from bacterial overgrowth and invasion, an important role in the pathogenesis of acute or chronic inflammatory hepatobiliary diseases has also been attributed to microbial products or metabolites. For example, the major cell wall component endotoxin from Gram-negative bacteria and lipoteichoic acid from Gram-positive bacteria promote acute and chronic inflammatory and profibrogenic responses, and trimethylamine-N-oxide, a gut microbiota-dependent metabolite, has been associated with the development and severity of non-alcoholic fatty liver disease and formation of gallstones [41–45]. In the present study, we detected corisin, a microbiota-derived peptide, in the bile of patients with obstructive biliary diseases. Corisin is a 19-amino acid peptide released by proteolytic degradation of bacterial transglycosylase that induces apoptosis of epithelial cells from different tissues [25, 46]. The corisin sequence is conserved in various species of staphylococci and other bacterial strains, including strains of *Listeria monocytogenesis,* an agent of food poisoning, and *Mycobacterium abscessus*, an organism often associated with lung infections [25]*.* Previous reports have demonstrated that corisin is involved in acute lung injury and acute exacerbation of pulmonary fibrosis [24–26]. The detection of corisin in bile from patients with biliary obstruction suggests that corisin may also play a role in the pathogenesis of hepatobiliary disorders.

Acute cholangitis (AC) is a life-threatening condition that requires early diagnosis and treatment to prevent systemic complications and reduce mortality [1, 47]. The Tokyo Guidelines, established by a panel of international experts, have improved diagnostic yield and facilitated decision-making in the early stages of the disease by recommending a combination of clinical, laboratory, and imaging findings [6, 29, 30]. However, there are still cases of AC that are difficult to diagnose and manage [13, 48]. Therefore, further studies have been conducted to develop new biomarkers to detect severe AC cases requiring immediate therapeutic interventions. One of the potential biomarkers is procalcitonin, a calcitonin precursor produced by the thyroid gland, which has been shown to predict the severity of AC, sepsis, and the need for antibiotics and urgent biliary drainage [49–53]. Other blood biomarkers that have been reported to predict the severity of AC include presepsin, a fragment of the CD14 receptor complex; lactate, a metabolic product of host cells and bacteria; and lipocalin 2, a transporter of small and hydrophobic molecules [54–56]. In this study, we found that the plasma level of corisin, a novel peptide of bacterial origin, was significantly elevated in patients with AC, particularly those with severe disease, compared to non-severe and non-AC patients. Moreover, the plasma corisin level was significantly associated with the clinical grade of AC in the multivariate analysis and showed high specificity in detecting AC severity in the ROC curve analysis. These observations suggest that plasma corisin level may be a valuable predictor of disease severity in AC patients. However, the weak association of plasma corisin with the clinical grade of disease in the univariate analysis and its poor sensitivity to detect disease severity in the ROC curve analysis indicate that plasma corisin alone may not be sufficient for diagnosing AC severity and that it may need to be combined with other variables to improve its diagnostic performance. In addition to plasma biomarkers, bile components may provide useful information for diagnosing biliary tract diseases. Recent investigations have explored the possibility of using specific bile salts, proteins, nucleic acids, fatty acids, or microbiome-derived products as biomarkers [34]. In this context, we found that the bile level of corisin was significantly elevated in AC patients with severe disease compared to non-severe AC patients. Furthermore, the bile corisin level was significantly correlated with the clinical grade of AC severity and with blood markers of inflammation, coagulation, and renal function impairment in the univariate analysis. The bile corisin level also showed significant performance for diagnosis of disease severity in the ROC analysis. These results suggest that bile corisin level may be a useful biomarker of the severity of AC and that corisin in bile may play a role in the pathophysiology of AC by modulating the inflammatory, coagulation, and renal pathways.

The main limitations of this study are the single-center design, the high proportion of elderly patients, the lack of homogeneous backgrounds of the study groups, and the small sample size, which may restrain the generalizability of the results to the global population and other age groups.

## Conclusion

In this study, we demonstrated elevated levels of corisin, a microbiome-derived pro-apoptotic peptide, in both the plasma and bile of patients with AC. Furthermore, we found a strong correlation between corisin levels in bile and the severity of the disease, as well as its association with inflammatory response, coagulation activation, and renal dysfunction. Additionally, independent of other variables, we observed a significant relationship between plasma corisin levels and disease severity in AC patients. These findings collectively indicate the potential value of measuring plasma and bile corisin levels as a biomarker for AC. Nevertheless, further investigations are essential to validate the diagnostic value of corisin in AC, elucidate its underlying molecular mechanisms, and explore its potential as a therapeutic target.

### Supplementary Information

**Additional file 1: Figure S1.** Genus abundance in bile samples. The DNA was extracted from the bile samples, and 16S rDNA sequencing, microbial composition analysis, assessment of microbial diversity, and taxonomic assignment were as described under Materials and Methods. Non-AC, non-acute cholangitis patients; AC, acute cholangitis patients. **Table S1.** Clinical and laboratory profile of all subjects grouped by cause of biliary obstruction.

## Data Availability

All data generated or analyzed during the current study are included in the article. Also, any data and materials are available from the corresponding authors upon reasonable request.
