# Supplementary material for: Elevated plasma and bile levels of corisin, a microbiota-derived proapoptotic peptide, in patients with severe acute cholangitis
Source: Gut Pathog. 2023 Nov 30;15:59. doi: 10.1186/s13099-023-00587-4 (PMC10688013; doi:10.1186/s13099-023-00587-4)
Supplement: Supplementary file 1 — Additional file 1: Figure S1. Genus abundance in bile samples. The DNA was extracted from the bile samples, and 16S rDNA sequencing, microbial composition analysis, assessment of microbial diversity, and taxonomic assignment were as described under Materials and Methods. Non-AC, non-acute cholangitis patients; AC, acute cholangitis patients. Table S1. Clinical and laboratory profile of all subjects grouped by cause of biliary obstruction. [file 13099_2023_587_MOESM1_ESM.docx]

**Elevated Plasma and Bile Levels of Corisin, a Microbiota-Derived Proapoptotic Peptide, in Patients with Severe Acute Cholangitis**

Ryo Nishiwaki, Ichiro Imoto, Satoko Oka, Taro Yasuma, Hajime Fujimoto, Corina N. D’Alessandro-Gabazza, Masaaki Toda, Tetsu Kobayashi, Hataji Osamu, Kodai Fujibe, Kenichiro Nishikawa, Tetsuya Hamaguchi, Natsuko Sugimasa, Midori Noji, Yoshiyuki Ito, Kenji Takeuchi, Isaac Cann, Yasuhiro Inoue, Toshio Kato, Esteban C Gabazza.


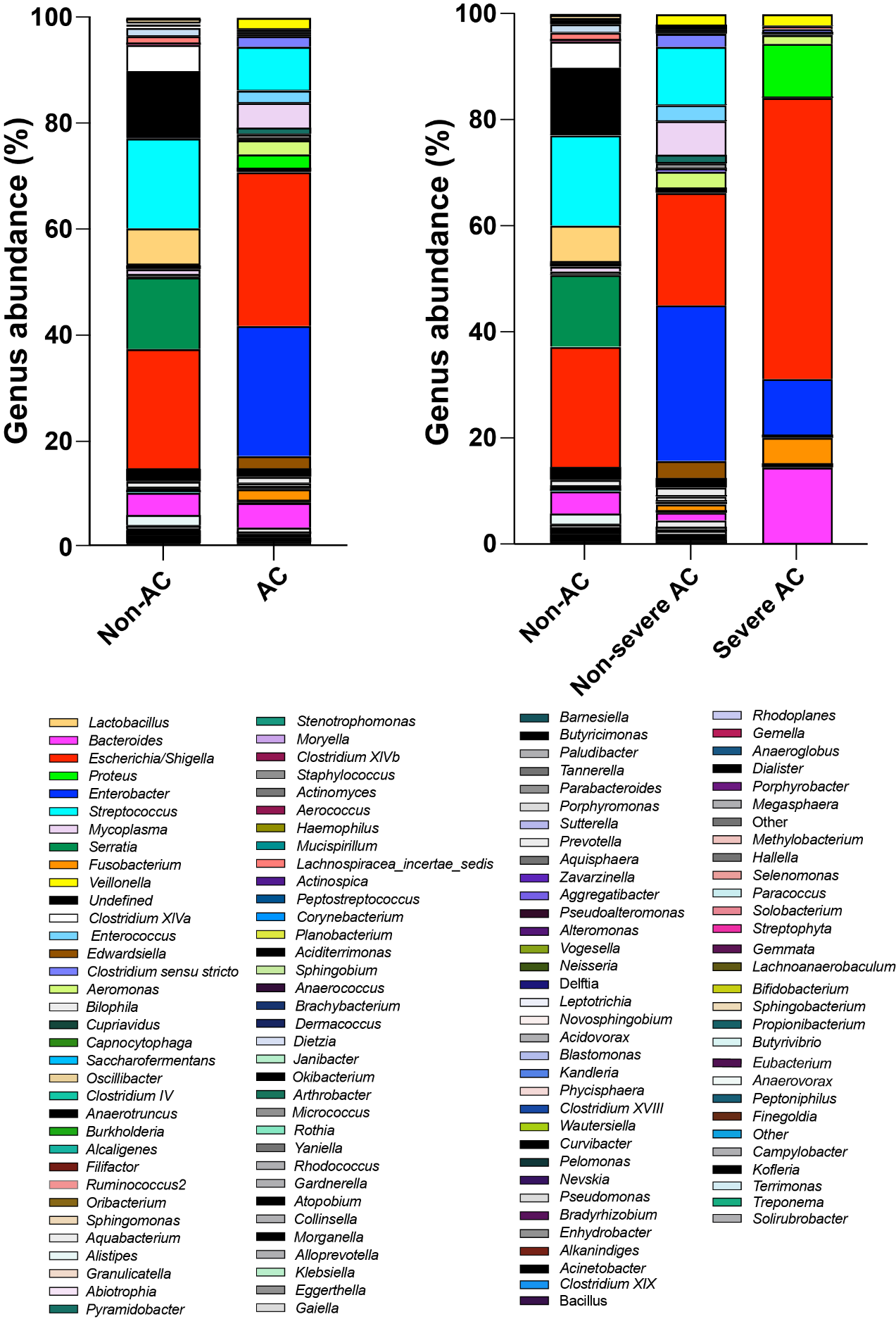


**Figure S1. Genus abundance in bile samples.** The DNA was extracted from the bile samples, and 16S rDNA sequencing, microbial composition analysis, assessment of microbial diversity, and taxonomic assignment were as described under Materials and Methods. Non-AC, non-acute cholangitis patients; AC, acute cholangitis patients.

| **Table S1. Clinical and laboratory profile of all subjects grouped by cause of biliary obstruction** | | | | | |
| --- | --- | --- | --- | --- | --- |
|  | Lemmel syndrome | Lithiasis＋Malignant tumor | Lithiasis | Malignant tumor | ANOVA p values |
| No of subjects | 1 | 1 | 29 | 9 |  |
| Systolic pressure (mmHg) | 120.00 | 141.00 | 127.03 ± 4.39 | 133.22 ± 6.30 | 0.10 |
| Diastolic pressure (mmHg) | 76.00 | 71.00 | 69.06 ± 2.49 | 69.00 ± 3.38 | 0.06 |
| Body temperature (Celcius) | 37.08 | 37.10 | 36.92 ± 0.14 | 36.58 ± 0.13 | 0.25 |
| White blood cells (103/µL) | 6.30 | 22.70 | 10.53 ± 0.92 | 8.37 ± 1.22 | 0.66 |
| Blood platelets (104/µL) | 102.00 | 333.00 | 176.79 ± 15.80 | 175.55 ± 16.95 | 0.37 |
| Blood hemoglobin (g/dL) | 11.70 | 11.60 | 12.23 ± 0.34 | 9.90 ± 0.82 | 0.66 |
| Blood total protein (g/dL) | 5.50 | 7.00 | 6.51 ± 0.12 | 6.32 ± 0.29 | 0.23 |
| Blood albumin (g/dL) | 3.10 | 2.80 | 3.21 ± 0.12 | 2.76 ± 0.27 | 0.24 |
| Blood total bilirubin (mg/dL) | 4.32 | 1.57 | 3.26 ± 0.51 | 3.93 ± 0.77 | 0.11 |
| Blood direct bilirubin (mg/dL) | 2.53 | 0.79 | 1.92 ± 0.50 | 2.47 ± 0.85 | 0.08 |
| Serum aspartate aminotransferase (U/L) | 716.00 | 29.00 | 216.34 ± 45.70 | 127.88 ± 21.93 | 0.54 |
| Serum alanine transaminase (U/L) | 233.00 | 32.00 | 173.20.1 ± 29.49 | 102.22 ± 13.73 | 0.22 |
| Serum γ-glutamyl transpeptidase (U/L) | 97.00 | ND | 336.51 ± 51.05 | 563.66 ± 134.55 | 0.40 |
| Serum alkaline phosphatase (U/L) | 77.00 | 413.00 | 289.51 ± 41.53 | 584.55 ± 134.52 | 0.65 |
| Serum amylase (U/L) | 69.00 | 40.00 | 71.75 ± 9.72 | 124.44 ± 39.99 | 0.31 |
| Serum Na (mmol/L) | 141.00 | 131.00 | 137.58 ± 0.80 | 137.33 ± 1.20 | 0.25 |
| Serum K (mmol/L) | 3.30 | 4.20 | 3.97 ± 0.11 | 4.25 ± 0.19 | 0.25 |
| Serum Cl (mmol/L) | 109.00 | 94.00 | 99.01 ± 3.29 | 103.44 ± 1.16 | 0.10 |
| Serum procalcitonin | 6.70 | 2.40 | 4.96 ± 1.70 | 8.02 ± 7.28 | 0.07 |
| Serum C-reactive protein (mg/dL) | 5.40 | 33.49 | 9.70 ± 1.93 | 8.59 ± 3.09 | 0.44 |
| Blood urea nitrogen (mg/dL) | 22.90 | 9.70 | 26.75 ± 4.35 | 26.12 ± 3.56 | 0.08 |
| Serum creatinine (mg/dL) | 1.03 | 0.35 | 1.45 ± 0.28 | 1.79 ± 0.69 | 0.09 |
| Activated partial thromboplastin time (sec) | 43.40 | 50.20 | 35.69 ± 1.79 | 33.75 ± 2.12 | 0.31 |
| Prothrombin time (sec) | 30.00 | 64.00 | 81.81 ± 5.50 | 85.77 ± 5.16 | 0.35 |
| Plasma fibrin-degradation products (µg/mL) | 3.70 | 0.00 | 14.00 ± 2.91 | 22.30 ± 10.56 | 0.18 |
| Peripheral oxygen saturation (%) | 97.00 | 95.00 | 97.03 ± 0.29 | 95.66 ± 1.50 | 0.20 |
| Plasma IL-6 (pg/mL) | 166.21 | 498.15 | 2620.86 ± 1100.87 | 5355.25 ± 48.53.06 | 0.10 |
| Plasma TNFα (pg/mL) | 274.90 | 264.29 | 452.51 ± 75.46 | 955.57 ± 428.11 | 0.31 |
| Plasma FasL (pg/mL) | 81.43 | 73.69 | 73.97 ± 8.91 | 65.78 ± 4.94 | 0.06 |
| Plasma corisin (pg/ml) | 656.69 | 2512.40 | 1473.69 ± 307.33 | 1115.73 ± 259.75 | 0.12 |
| Bile IL-6 (pg/mL) | 136.99 | 281.47 | 546.39 ± 185.97 | 1776.46 ± 1123.45 | 0.26 |
| Bile TNFα (pg/mL) | 198.83 | 709.48 | 560.08 ± 117.36 | 1054.53 ± 365.87 | 0.26 |
| Bile FasL (pg/mL) | 70.46 | 62.27 | 123.72 ± 16.76 | 183.11 ± 79.16 | 0.16 |
| Bile corisin (pg/ml) | 878.15 | 855.85 | 1064.54 ± 157.52 | 1330.35 ± 321.76 | 0.09 |
| Data are expressed as the mean ±standard error of the mean. TNF-α, tumor necrosis factor-α; IL-6, interleukin-6. ANOVA, analysis of variance. | | | | | |

| **Supplementary Table 2. Microbial etiology of acute cholangitis** | | | |
| --- | --- | --- | --- |
|  |  | No of cases | % |
| Total number of acute cholangitis cases | | 40 | 100 |
| Bile culture (+) | | 30 | 75 |
|  | *Escherichia coli* | 24 | 60 |
|  | *Klebsiella pneumoniae ssp pneumoniae* | 18 | 45 |
|  | *Enterococcus spp* | 13 | 32.5 |
|  | *Clostridium perfringens* | 5 | 12.5 |
|  | *Aeromonas hydrophila/punctata* | 4 | 10 |
|  | *Citrobacter freundii* | 3 | 7.5 |
|  | *Enterococcus avium* | 3 | 7.5 |
|  | *Bacteroides fragilis* | 2 | 5 |
|  | *Enterococcus faecalis* | 2 | 5 |
|  | *Enterococcus faecium* | 2 | 5 |
|  | *Klebsiella oxytoca* | 2 | 5 |
|  | *Streptococcus anginosus* | 2 | 5 |
|  | *Klebsiella pneumoniae ssp ozaenae* | 1 | 2.5 |
|  | *Citrobacter farmeri* | 1 | 2.5 |
|  | *Citrobacter amalonaticus* | 1 | 2.5 |
|  | *Enterobacter intermedius* | 1 | 2.5 |
|  | *Edwardsiella tarda* | 1 | 2.5 |
|  | *Proteus mirabilis* | 1 | 2.5 |
|  | *Morganella morganii* | 1 | 2.5 |
|  | *Pseudomonas aeruginosa* | 1 | 2.5 |
|  | *Raoultella planticola* | 1 | 2.5 |
|  | *γ-Streptococcus* | 1 | 2.5 |
|  | *Candida albicans* | 1 | 2.5 |
|  | *Streptococcus parasanguinis* | 1 | 2.5 |
| Bile culture (-) | | 10 | 25 |
| Patients with mixed microbial infection | | 25 | 62.5 |
| Patients with single microbial infection | | 5 | 12.5 |
|  | | | |
